# Conjugate addition–enantioselective protonation reactions

**DOI:** 10.3762/bjoc.12.116

**Published:** 2016-06-15

**Authors:** James P Phelan, Jonathan A Ellman

**Affiliations:** 1 Department of Chemistry, Yale University, 225 Prospect Street, New Haven, CT 06520, USA

**Keywords:** asymmetric catalysis, conjugate addition, enantioselective protonation, enolate

## Abstract

The addition of nucleophiles to electron-deficient alkenes represents one of the more general and commonly used strategies for the convergent assembly of more complex structures from simple precursors. In this review the addition of diverse protic and organometallic nucleophiles to electron-deficient alkenes followed by enantioselective protonation is summarized. Reactions are first categorized by the type of electron-deficient alkene and then are further classified according to whether catalysis is achieved with chiral Lewis acids, organocatalysts, or transition metals.

## Introduction

Due to their ubiquity in natural products and drugs, many researchers have developed methods for the stereoselective synthesis of tertiary carbon stereocenters. One aesthetically pleasing approach is the enantioselective protonation of prochiral enolates and enolate equivalents [1–10]. While an attractive strategy, the enantioselective introduction of a proton, the smallest element in the periodic table, presents its own unique challenges. Significant racemic background reactions can compete with the desired enantioselective protonation because proton transfer is among the fastest of all processes. The α-electron withdrawing group, needed to stabilize the carbanion intermediate, also increases the stereocenter’s susceptibility to racemization under the reaction conditions. Moreover, enolate intermediates can adopt *E-* or *Z-*geometries that, upon protonation, generally lead to opposite stereoisomers.

**Figure 1:**
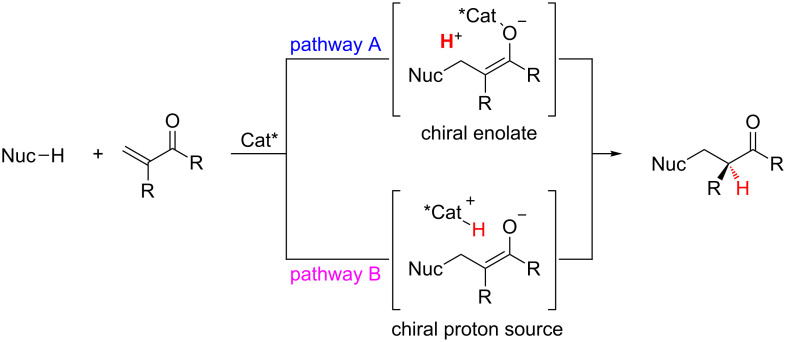
Two general pathways for conjugate addition followed by enantioselective protonation.

Because enantioselective protonation is a kinetic process, an overall thermodynamic driving force is required for any enantioselective protonation reaction [1]. One attractive approach is the coupling of the enantioselective protonation step with another bond forming step. Conjugation addition of an organometallic or protic nucleophile in a non-stereoselective step allows for the generation of a prochiral enolate intermediate that then undergoes enantioselective protonation (Figure 1). Two general strategies can be used when applying a conjugate addition–enantioselective protonation manifold. In the first strategy, a chiral enolate can be protonated by an achiral proton source (pathway A). In the second, an achiral enolate can be protonated by a chiral proton source (pathway B). Both strategies have been harnessed by various research groups for conjugate addition–enantioselective protonation reactions.

This review covers the many conjugate addition–enantioselective protonation reactions that have been reported in the literature. These reports have been grouped by class of Michael acceptor and further subdivided by the type of catalyst system used (Lewis acids, organocatalysts and transition metals). While numerous efficient methods have been developed for the enantioselective reduction of α-substituted conjugate addition acceptors, including catalytic hydrogenation, multiple reviews have already appeared on this topic, and therefore asymmetric catalytic reduction will not be covered here [11–13]. Conjugate addition followed by terminal enantioselective hydrogen atom transfer is an approach that provides products analogous to those accessed via conjugate addition–enantioselective protonation sequences. Due to the similar challenges when delivering both protons and hydrogen atoms enantioselectively, examples of conjugate addition–enantioselective hydrogen atom transfer are also included in this review.

## Review

### α,β-Unsaturated esters

#### Lewis acids

α,β-Unsaturated esters have been the most extensively studied class of electrophiles for conjugate addition–enantioselective protonation sequences. Esters are useful functional group handles for additional synthetic manipulations. Furthermore, enantioselective conjugate additions to α-amino-α,β-unsaturated esters provides rapid access to enantioenriched α-amino acid derivatives. However, α,β-unsaturated esters present some challenges; the transient enolate intermediate can adopt *E*- or *Z*-enolate geometries. Also, esters are not as electron deficient as many other electron withdrawing groups, and therefore, the presence of additional activating groups on the alkene or the use of a highly activating catalyst are often required.

**Scheme 1:**
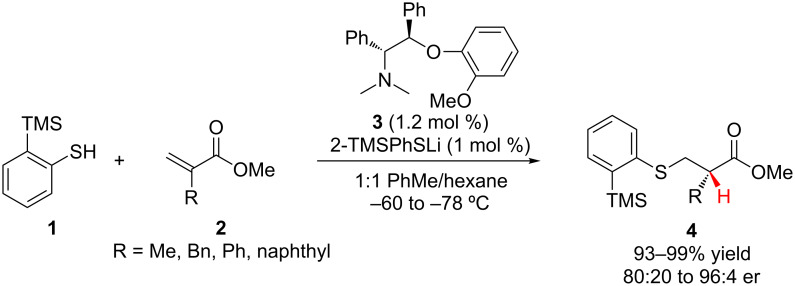
Tomioka’s enantioselective addition of arylthiols to α-substituted acrylates.

In 2001, Tomioka and co-workers reported the addition of lithium arylthiolates, catalytically generated from **1,** to α-substituted acrylates **2** followed by enantioselective protonation of the resulting lithium enolate (Scheme 1) [14]. The *ortho*-trimethylsilyl substituent on the phenyl ring was necessary for achieving high levels of enantioselectivity. All of the reactions proceeded in high yield, with the best enantioselectivities being observed for α-arylacrylates (94:6 to 96:4 er).

**Scheme 2:**
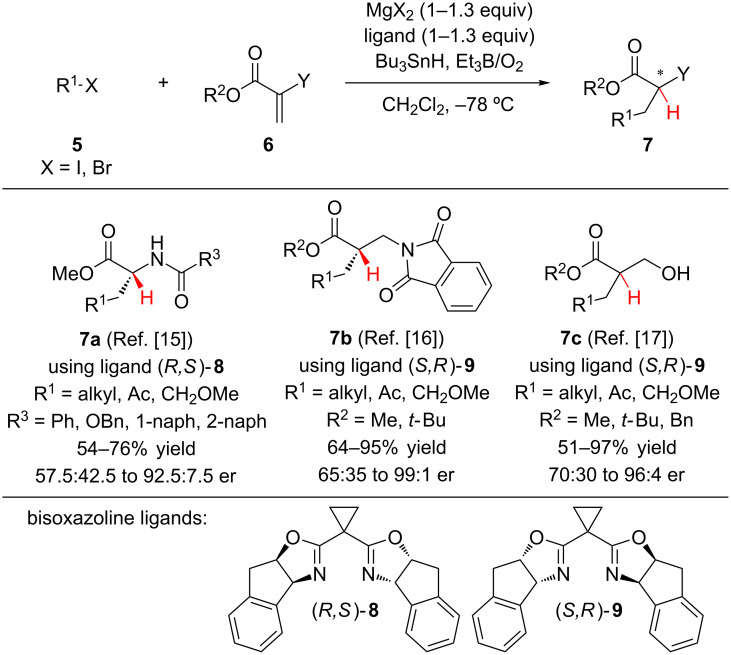
Sibi’s enantioselective hydrogen atom transfer reactions.

For the synthesis of enantioenriched α-amino esters **7a**, β-amino esters **7b** and 2-hydroxymethyl esters **7c**, the Sibi group has utilized a conjugate addition–enantioselective hydrogen atom transfer reaction (Scheme 2) [15–17]. A Mg-(bis)oxazoline complex serves as both a Lewis acid for the activation of α-substituted acrylates **6** towards radical addition and as a chiral template for an enantioselective hydrogen atom transfer from Bu_3_SnH to the α-ester radical intermediate. The authors found that the stoichiometric chiral Mg-(bis)oxazoline complex was required to achieve high enantioselectivity. Higher enantioselectivity was generally observed for reactions using secondary or tertiary alkyl halides. When performing radical conjugate additons to give 2-hydroxymethyl esters **7c**, the authors observed a complete turnover in the sense of induction based upon the ester substituent. The less bulky methyl and benzyl esters gave the (*S*)-enantiomer while the *tert*-butyl ester gave the (*R*)-enantiomer. The incorporation of aryl groups was not compatible with this reaction manifold, prompting Sibi to explore aromatic nucleophiles for the conjugate addition–enantioselective protonation of α-aminoacrylates (vide infra).

**Scheme 3:**
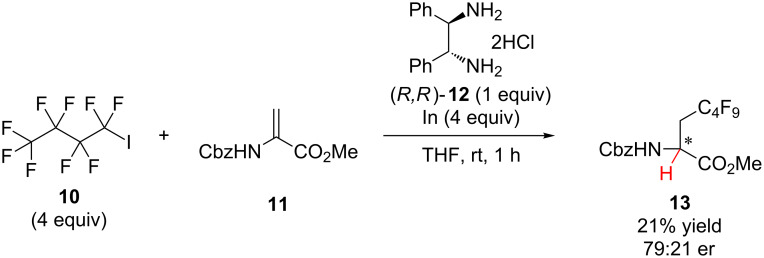
Mikami’s addition of perfluorobutyl radical to α-aminoacrylate **11**.

In 2010, Mikami and co-workers reported a hydrogen atom transfer strategy for the synthesis of β-perfluorobutyl-α-amino ester **13** in low yield and modest enantioselectivity, and the absolute configuration of the major enantiomer was not defined (Scheme 3) [18]. Indium was used to initiate the addition of a perfluorobutyl radical to α-aminoacrylate **11** followed by hydrogen atom transfer to the resulting α-amino α-ester radical from (*R,R*)-**12**.

**Scheme 4:**
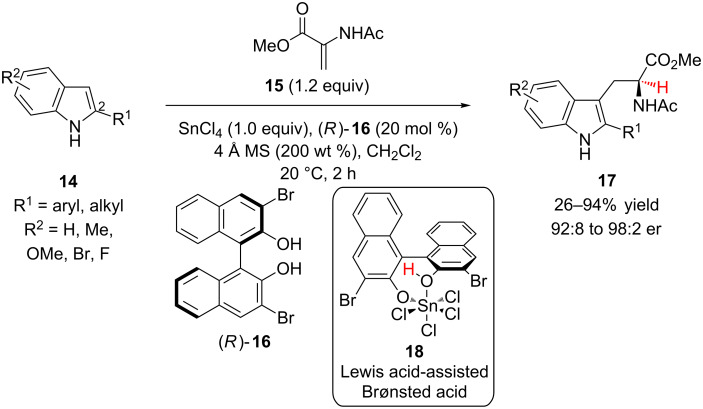
Reisman’s Friedel–Crafts conjugate addition–enantioselective protonation approach toward tryptophans.

Enantioenriched tryptophan derivatives are useful building blocks for the synthesis of biologically active molecules, including natural products and drugs. The Reisman group has reported a Friedel–Crafts conjugate addition–enantioselective protonation for the synthesis of tryptophans **17** from 2-substituted indoles **14** and methyl 2-acetamidoacrylate (**15**) using catalytic (*R*)-3,3’-dibromo-BINOL (**16**) and stoichiometric SnCl_4_ [19] (Scheme 4). The authors proposed that complex **18** acted as a chiral proton source to protonate a tin-enolate intermediate based upon related complexes that the Yamamoto group had previously used for the enantioselective protonation of silyl enol ethers [20–21]. Electron-rich and neutral indoles were efficient substrates for the reaction; however, electron-poor indoles showed attenuated reactivity even when 1.6 equivalents of SnCl_4_ were employed (60–63% yield, 96:4 to 96.5:3.5 er). Indoles lacking substitution at the 2-position (R^1^ = H) reacted in low yield and poor enantioselectivity. Sterically bulky 2-substituents (*ortho*-substituted phenyl, *tert*-butyl) showed attenuated reactivity but retained high enantioselectivity.

#### Organocatalysts

**Scheme 5:**
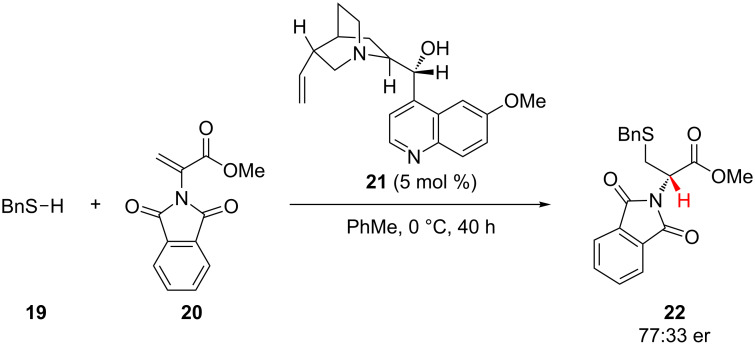
Pracejus’s enantioselective addition of benzylmercaptan to α-aminoacrylate **20**.

In a pioneering work from 1977 on conjugate addition–enantioselective protonation, Pracejus and co-workers explored the ability of chiral tertiary amines to catalyze the enantioselective addition of thiols to α-aminoacrylates (Scheme 5) [22]. The authors found that quinidine (**21**) catalyzed the enantioselective addition of benzylmercaptan (**19**) to α-aminoacrylate **20** in modest enantioselectivity. Acylation of the hydroxy group of quinidine resulted in complete loss of enantioselectivity, suggesting that hydrogen-bonding contacts between the catalyst’s hydroxy group and the substrate are important for organizing the transition state.

**Scheme 6:**
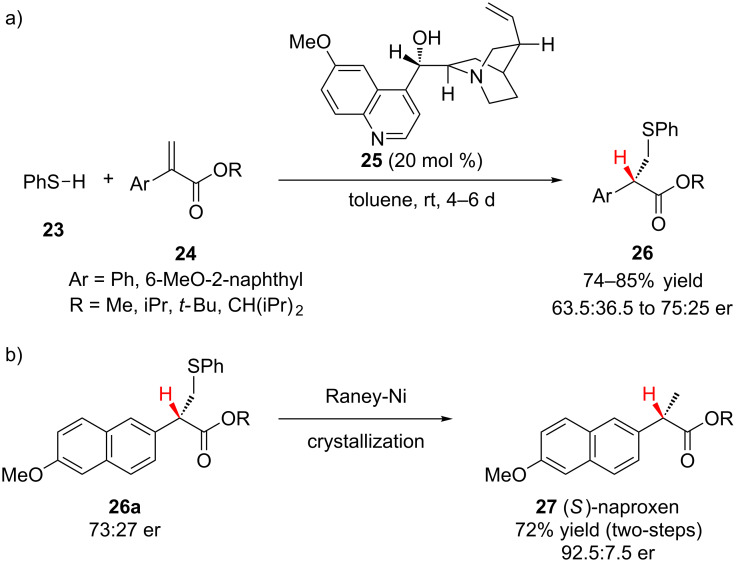
Kumar and Dike’s enantioselective addition of thiophenol to α-arylacrylates.

Using catalytic quinine (**25**) the pseudo-enantiomer of the quinidine catalyst employed by Pracejus, Kumar and Dike reported the enantioselective addition of thiophenol (**23**) to α-arylacrylates **24** in modest enantioselectivity (Scheme 6a) [23]. The authors found that sterically bulky esters (R = *t*-Bu, CH(iPr)_2_) negatively impacted the enantioselectivity of the reaction (63.5:36.5 to 67.5:32.5 er). To demonstrate the utility of the transformation, the sulfur–carbon bond of **26a** was reduced using Raney nickel to access (*S*)-naproxen (**27**), an anti-inflammatory drug (Scheme 6b).

**Scheme 7:**
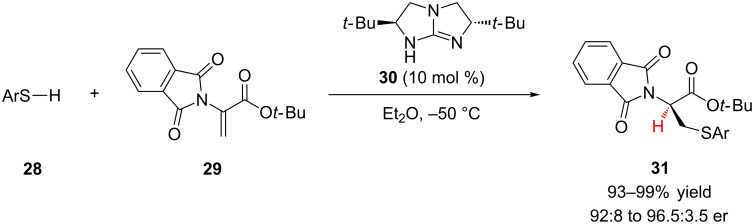
Tan’s enantioselective addition of aromatic thiols to 2-phthalimidoacrylates.

Inspired by the work of Pracejus, Tan and colleagues applied their *C*_2_-symmetric guanidine catalyst **30** to the enantioselective addition of aromatic thiols to 2-phthalimidoacrylates **29** in high yield and good enantioselectivity (Scheme 7) [24]. A variety of electron rich, neutral, and poor thiols coupled efficiently. Impressively, the aromatic thiol **28** could be substituted with hydroxy and amino groups without significantly affecting the reaction (93–97% yield, 92:8 to 96:4 er). The authors also explored further elaboration of the products to access enantioenriched cysteine analogues.

**Scheme 8:**
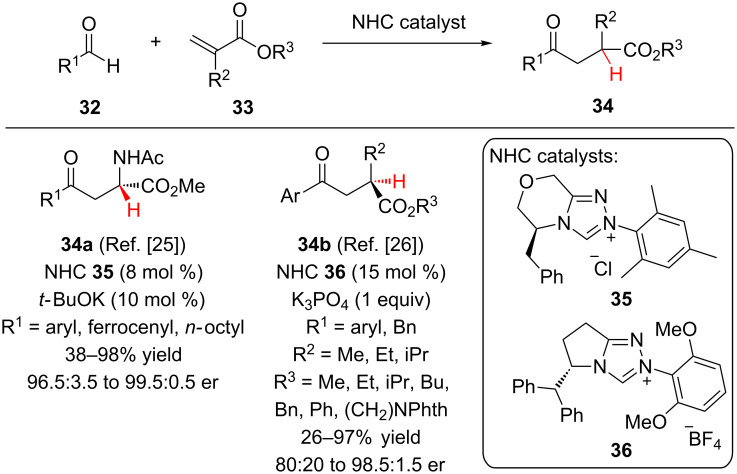
Glorius’ enantioselective Stetter reactions with α-substituted acrylates.

The Glorius lab has made use of N-heterocyclic carbene (NHC) catalysts for intermolecular Stetter reactions between aldehydes and α,β-unsaturated esters (Scheme 8) [25–26]. Catalyzed by triazolium NHC-catalyst **35**, electron-poor and neutral aromatic aldehydes reacted with methyl 2-acetamidoacrylate to access α-amino esters **34a** with excellent enantioselectivity [25]. As expected, less electrophilic 4-methoxybenzaldehyde led to low conversion. The use of potassium *tert*-butoxide was found to be essential to achieve good reactivity and enantioselectivity. Other amino-protecting groups (Boc, phthalimido) or tertiary *N*-methylated variants rendered the 1,4-acceptor unreactive. Glorius and co-workers proposed that an intramolecular proton transfer was the stereodetermining step. Alternatively, Sunoj and co-workers performed DFT calculations on the mechanism of the enantioselective Stetter reaction between *p*-chlorobenzaldehyde and *N*-acetylamido acrylate and proposed that *tert*-butyl alcohol plays a key role as the proton source in the stereodetermining step [27].

In 2012, Glorius and co-workers also reported the addition of aromatic aldehydes to α-carbon substituted acrylates **33** to provide differentiated 1,4-dicarbonyls **34b** with good enantioselectivity [26]. When optimizing the catalyst‘s structure, the authors found that NHC’s possessing a 2,6-dimethoxyphenyl moiety led to both superior reactivity and selectivity, with catalyst **36** being optimal. Catalyst **35** provided the product in less than 5% yield. It was proposed that a more electron-rich, and thereby more nucleophilic, NHC was needed because the α-carbon substituted acrylate substrates employed were less reactive than the previously utilized methyl 2-acetamidoacrylate. A variety of α-alkylacrylates were effective substrates, only when α-benzyl or α-phenylacrylates were used was there a decrease in enantioselectivity (80:20 to 90:10 er).

**Scheme 9:**
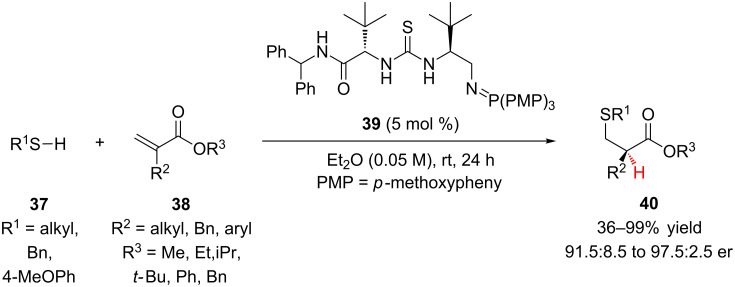
Dixon’s enantioselective addition of thiols to α-substituted acrylates.

Recently, Dixon reported the enantioselective conjugate addition of thiols to unactivated α-substituted acrylates followed by enantioselective protonation (Scheme 9) [28]. To overcome the low inherent electrophilicity of **38**, the authors postulated that bifunctional iminophosphoran (BIMP) organocatalysts with increased Brønsted basicity were required. Employing BIMP catalyst **39**, alkylthiols, phenylmethanethiol, and 4-methoxybenzenethiol were added to α-substituted acrylates in good yield and enantioselectivity. A variety of R^2^ and R^3^ substituents performed well under the reaction conditions, only for the sterically bulky *tert-*butyl ester was the reactivity significantly impacted (R^2^ = *n*-Pr, R^3^ = *t*-Bu, 36% yield, 98:2 er).

#### Transition metals

**Figure 2:**
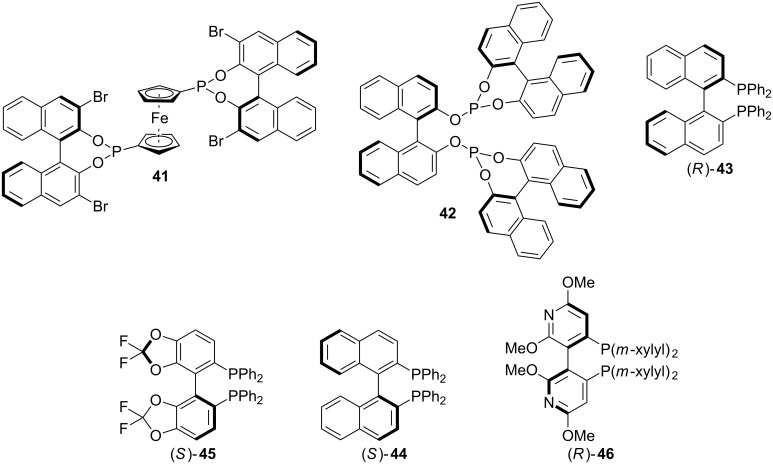
Chiral phosphorous ligands.

**Scheme 10:**
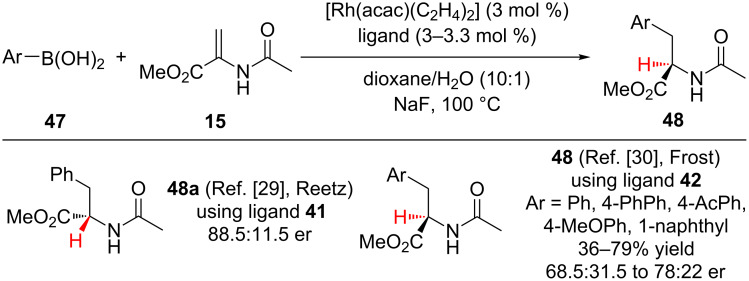
Enantioselective addition of arylboronic acids to methyl α-acetamidoacrylate.

A handful of research groups have investigated transition metal-catalyzed conjugate addition–enantioselective protonation sequences involving α,β-unsaturated esters. A common theme has been the use of rhodium(I) transition metal catalysts and axially chiral phosphorous ligands (Figure 2). Additionally, because organometallic reagents are often utilized as nucleophiles, an exogenous proton source, which can impact the transformation’s enantioselectivity, is frequently needed. In this context, Reetz and co-workers were the first to report the transition metal-catalyzed enantioselective addition of arylboronic acids to an α-substituted-α,β-unsaturated ester to provide enantioenriched phenylalanine derivatives **48a** (Scheme 10) [29]. Notably, a BINAP-derived rhodium(I) catalyst was superbly active (100% conversion) but provided a completely racemic product. Only by utilizing a less electron-rich diphosphonite ligand **41** was enantioinduction achieved. Building on the initial report from Reetz, Frost and colleagues identified diphosphite **42** as a competent ligand for the transformation (Figure 2), accessing a handful of phenylalanine analogues **48** in moderate yield and enantioselectivity (Scheme 10) [30].

**Scheme 11:**
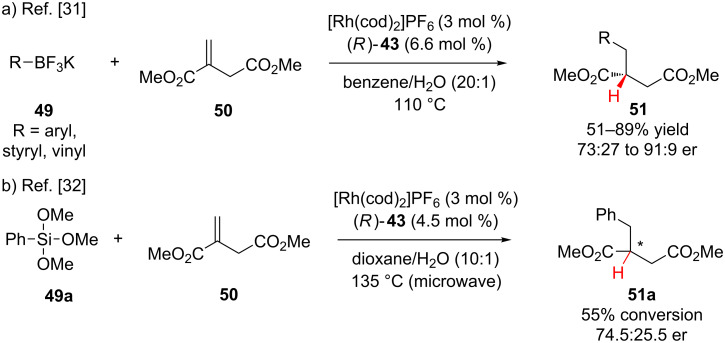
Frost’s enantioselective additions to dimethyl itaconate.

As shown in Scheme 11a, Frost and co-workers have also investigated conjugate addition–enantioselective protonation by the addition of potassium organotrifluoroborates **49** into dimethyl itaconate (**50**) in the presence of a rhodium(I) catalyst and (*R*)-BINAP ligand (**43**, Figure 2) [31]. During optimization they found that switching to potassium organotrifluoroborates from organoboronic acids was necessary to achieve high enantioinduction. Additionally, the enantioselectivity was highly dependent on the solvent system; the enantioselectivity in benzene was significantly higher than in toluene or dioxane. Electron rich, neutral, and poor organotrifluoroborates were good substrates; however, *ortho*-substitution was not compatible and provided only trace product. In a subsequent publication, Frost reported that using the same catalyst system, phenyltrimethoxysilane (**49a**) could be added to dimethyl itaconate (**50**) with modest enantioselectivity and without defining the absolute configuration of the major stereoisomer (Scheme 11b) [32].

**Scheme 12:**
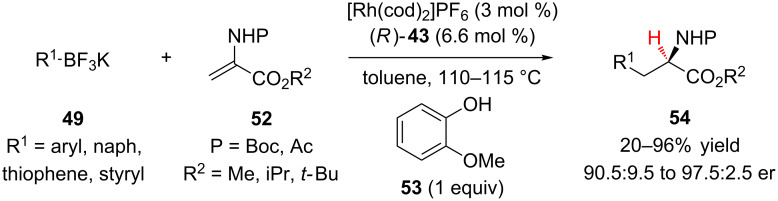
Darses and Genet’s addition of potassium organotrifluoroborates to α-aminoacrylates.

Darses and Genet reported the highly enantioselective Rh-catalyzed addition of potassium organotrifluoroborates **49** to α-aminoacrylates **52** to access phenylalanine derivatives **54** (Scheme 12) [33]. The authors identified guaiacol (**53**) as the optimal proton source, observing the general trend that less acidic phenols led to an increase in enantioselectivity. A wide range of aryltrifluoroborates were efficient substrates for the reaction to provide the products in good yield and enantioselectivity. Increasing the steric bulk of the ester (R^2^ = iPr, *t*-Bu) decreased the yield of the reaction but did not impact its enantioselectivity.

**Scheme 13:**
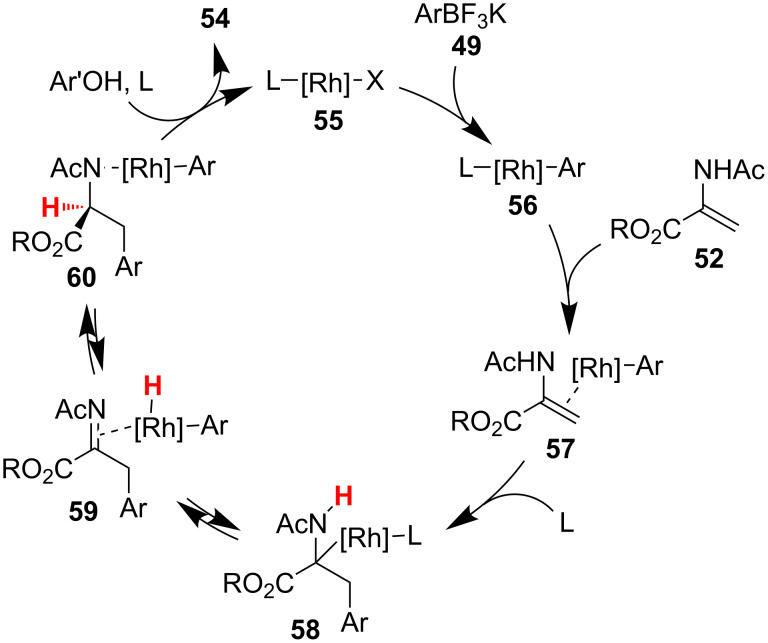
Proposed mechanism for enantioselective additions to α-aminoacrylates.

Darses and Genet probed the mechanism of their reaction through a combination of DFT calculations, deuterium labeling studies, and control experiments (Scheme 13) [34]. The authors proposed that after migratory insertion of the rhodium–aryl bond across the acrylate, **58** undergoes β-hydride elimination of the enamide proton to generate *N-*acylimine **59**. Addition of the rhodium-hydride across the imine is then the enantiodetermining step, followed by protodemetalation to generate the product **54**.

**Scheme 14:**
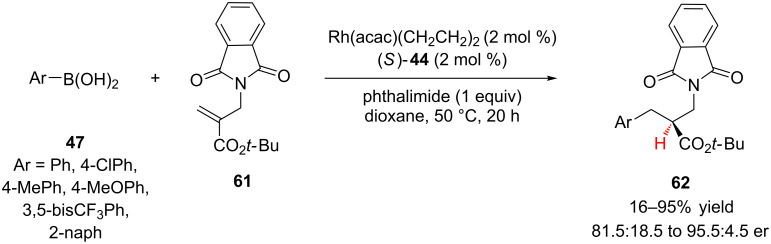
Sibi’s addition of arylboronic acids to α-methylaminoacrylates.

Inspired by previous reports on the synthesis of α-amino esters using rhodium(I) catalysis, Sibi and co-workers investigated the enantioselective synthesis of β-amino esters **62** via the addition of arylboronic acids **47** to α-methylaminoacrylates **61** (Scheme 14) [35]. The authors identified (*S*)-difluorphos (**44**, Figure 2) and phthalimide as the optimal ligand and proton source combination, respectively. Additionally, the bulky *tert*-butyl ester was necessary for achieving both good reactivity and enantioselectivity. All of the arylboronic acids investigated, except 4-methylphenylboronic acid, added in good yield and enantioselectivity (70–95% yield, 92:8 to 95.5:4.5 er).

**Scheme 15:**
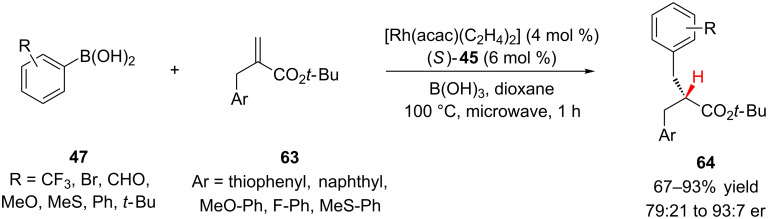
Frost’s enantioselective synthesis of α,α-dibenzylacetates **64**.

In 2007, Frost and co-workers reported a rhodium catalyzed conjugate addition–enantioselective protonation to prepare esters with α,α-dibenzyl substitution [36]. The addition of arylboronic acids **47** to α-benzylacrylates **63** were catalyzed by a rhodium(I) (*S*)-BINAP (**45**, Figure 2) complex with boric acid as a proton source (Scheme 15). A variety of electron-rich, neutral, and electron-poor arylboronic acids added in good yields and enantioselectivity to access esters with α,α-dibenzyl substitution and with only subtle steric and electronic differences between the two benzyl groups.

**Scheme 16:**
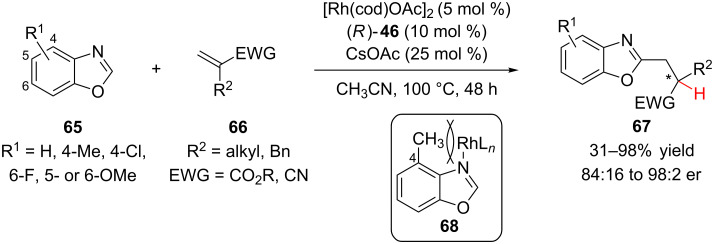
Rovis’s hydroheteroarylation of α-substituted acrylates with benzoxazoles.

Recently, Rovis and co-workers reported a detailed study on the enantioselective hydroheteroarylation of α-substituted acrylates **66** with benzoxazoles **65** in moderate to good yields and good to excellent enantioselectivity (Scheme 16) [37]. The absolution configuration of the major enantiomer was not determined. The authors found that sterically encumbered bisphosphine ligand **46** (Figure 2) was necessary for achieving high reactivity, presumably because it minimizes undesired ligation of the benzoxazole substrates or intermediates. A variety of α-substituted acrylates as well as methacrylonitrile were good substrates. The system is sensitive to sterics, an acetate additive was needed to improve the reactivity of the more hindered acrylate substrates (e.g., R^2^ = Bn) and when R^2^ was phenyl or isopropyl no reactivity was observed. Substituted benzoxazoles were also effective with 4-methylbenzoxazole being a preferred substrate. The authors proposed that substitution at the 4-position disfavored rhodium catalyst binding to the benzoxazole nitrogen **68**.

**Scheme 17:**
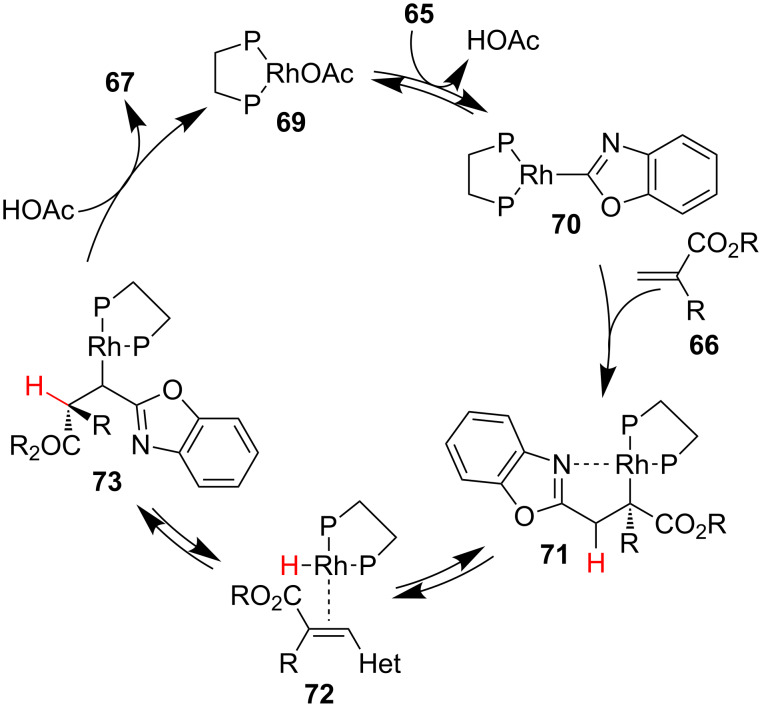
Proposed mechanism for the hydroheteroarylation of α-substituted acrylates with benzoxazoles.

Deuterium labeling studies were performed on the system and based on their results a mechanism was proposed in which the stereodetermining step is a rhodium-hydride transfer instead of protonation of an oxo-π-allylrhodium species (Scheme 17). Insertion into the C–H bond of **65** provides intermediate **70**, which then undergoes migratory insertion into acrylate **66** to give **71**. β-Hydride elimination to give the α,β-disubstituted acrylate **72** followed by enantioselective hydride transfer generates the chiral tertiary center in **73**. Finally, protodemetalation liberates the product and regenerates **69**. While the overall process provides the same product as a conjugate addition–enantioselective protonation sequence, mechanistically this, along with other transition metal-catalyzed reactions that invoke a conjugate addition–enantioselective protonation manifold might possibly operate via different pathways.

### α,β-Unsaturated imides

#### Lewis acids

The catalytic generation of enolates in situ, while aesthetically attractive, presents its own set of challenges due to the potential for both *E*- and *Z*-enolate isomers that in some cases equilibrate under the reaction conditions. In this context, α,β-unsaturated imides are attractive substrates for conjugate addition–enantioselective protonation sequences. Imides are not only more electron withdrawing than esters, but the presence of a second carbonyl also provides an additional point of contact through which the chiral catalyst can bind and organize the transition state.

**Scheme 18:**
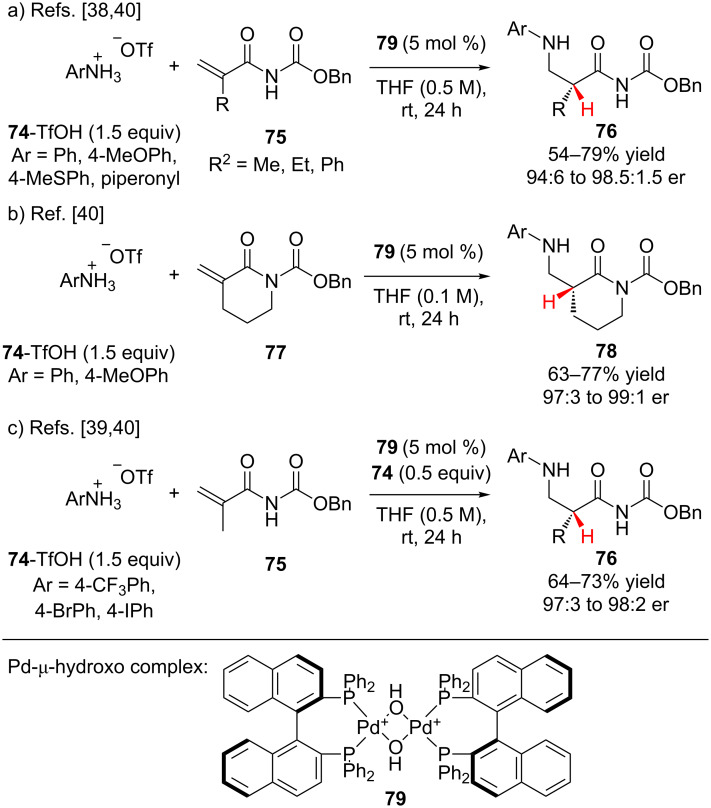
Sodeoka’s enantioselective addition of amines to *N*-benzyloxycarbonyl acrylamides **75** and **77**.

Many examples of conjugate addition–enantioselective protonation have been reported using carbon and sulfur nucleophiles, conversely relatively few examples have been reported using amines as nucleophiles. Sodeoka and co-workers have described the synthesis of β-aminocarbonyl compounds via the enantioselective addition of amine salts **74** to *N*-benzyloxycarbonyl acrylamides **75** and **77** catalyzed by a palladium-μ-hydroxo complex **79** (Scheme 18) [38–40]. Hii has used the same catalyst system and reported comparable yields and slightly lower enantioselectivities for the addition of *para*-substituted anilines to α,β-unsaturated imines [41]. Gil and Collin have also reported on the same reaction as Sodeoka, but using a samarium-BINOL catalyst system, which proceeded with lower enantioselectivity [42]. Sodeoka found that minimizing the concentration of free amine present in the reaction mixture by using the triflate salts of the amines was crucial for obtaining high enantioselectivity. Excess free amine resulted in catalyst deactivation and a racemic background reaction. Electron-rich and neutral aromatic amines added to both acyclic and cyclic *N*-benzyloxycarbonyl acrylamides **75** and **77** in moderate to good yields and high enantioselectivity (Scheme 18a,b). Due to their attenuated nucleophilicity, electron-deficient amines were unreactive under the standard reaction conditions. Addition of 0.5 equivalents of free amine was found to be optimal, providing the product with excellent enantioselectivity (Scheme 18c). Notably, the authors did not observe any side reaction between the palladium and aryl bromide or aryl iodide groups.

**Scheme 19:**
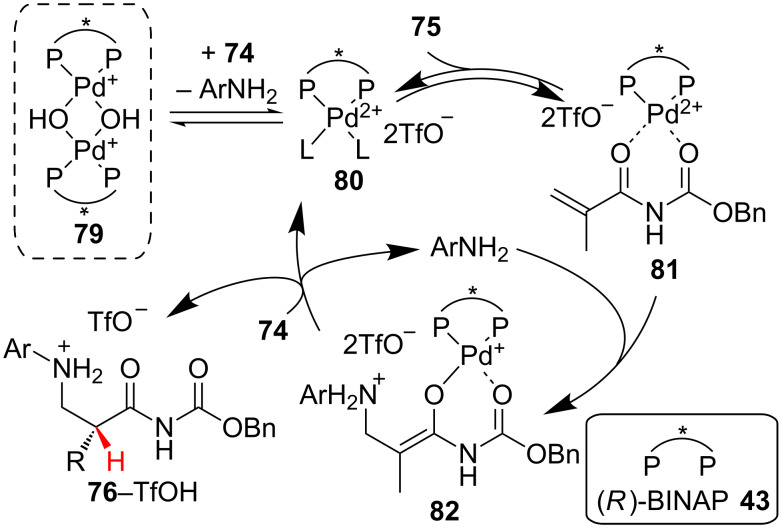
Proposed catalytic cycle for Sodeoka’s enantioselective addition of amines.

Using NMR analysis and ESIMS Sodeoka and co-workers probed the mechanism of the reaction, observing a complex interplay between ligand exchange and the ammonium salt (Scheme 19) [40]. Based on their findings they proposed a catalytic cycle in which the Brønsted basic dimeric palladium-μ-hydroxo complex **79** is broken up by the amine salt **74**, liberating Lewis acidic palladium monomer **80** and free amine. Binding of the *N*-benzyloxycarbonyl acrylamide by **80** activates it for conjugate addition of the amine to generate chiral enolate **82**. Enantioselective protonation of chiral enolate **82** by a second equivalent of amine salt liberates the product as the triflate salt and regenerates **80** and the free amine.

**Scheme 20:**
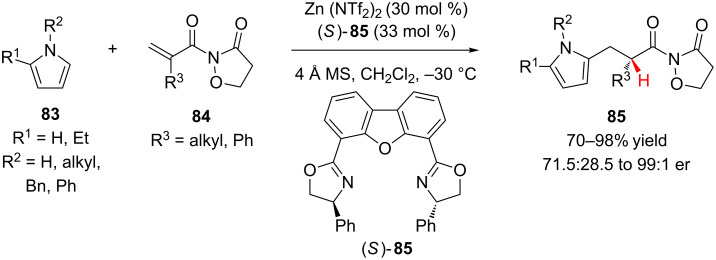
Sibi’s enantioselective Friedel–Crafts addition of pyrroles to imides **84**.

Building upon their previous work on enantioselective additions to α-substituted acrylates (vide ante), Sibi and colleagues reported the first example of Friedel–Crafts alkylation of an α-substituted-α,β-unsaturated imide followed by enantioselective protonation (Scheme 20) [43]. Using an in situ generated complex formed from Zn(NTf_2_)_2_ and Ph-dbfox ligand (*S*)-**85**, pyrroles **83** were added to imides **84** to produce **85** in high yield and modest to excellent enantioselectivity. Exploring a number of achiral imides, the authors found that an isoxazolidinone auxiliary enhanced the reactivity and minimized enolate A^1,3^-interactions to provide the best yield and enantioselectivity. *N*-Alkylpyrroles were effective substrates, adding with high enantioselectivity (94:6 to 99:1 er). The enantioselectivity of the transformation was significantly lower only for pyrroles that lacked substitution on nitrogen and for *N*-phenylpyrroles (R^2^ = H, Ph 71.5:28.5 to 90.5:9.5 er).

**Scheme 21:**
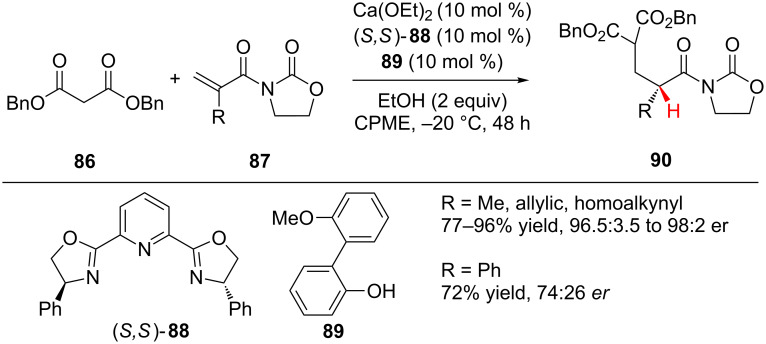
Kobayashi’s enantioselective addition of malonates to α-substituted *N*-acryloyloxazolidinones.

In 2010, the Kobayashi group reported the calcium-catalyzed Michael addition of dibenzylmalonate (**86**) to *N-*acryloyloxazolidinones **87** followed by enantioselective protonation of a chiral calcium enolate to access 1,5-dicarbonyl compounds **90** (Scheme 21) [44]. The chiral calcium alkoxide complex was generated in situ by mixing Ca(OEt)_2_, PyBox ligand (*S,S*)-**88**, and phenol **89**. The authors found that the slow addition of malonate **86** and the use of phenol **89** and ethanol as additives were all crucial for achieving high yield and enantioselectivity, although the exact role of the phenol was not elucidated. 1,5-Dicarbonyl compounds **90** were accessed with excellent enantioselectivity for a variety of aliphatic α-substituents, and only when R was phenyl was there a significant loss of enantioselectivity (72% yield, 74:26 er).

#### Organocatalysts

Multiple groups have developed organocatalytic conjugate addition–enantioselective protonations of α,β-unsaturated imides using thiols. Thiols are attractive nucleophiles due to their acidity, which facilitates deprotonation by amine bases and also reduces undesired competitive deprotonation and epimerization of the enolizable conjugate addition products. Additionally, the thiolate conjugate bases are highly nucleophilic, allowing ready access to sulfur functionalized products. A common theme among the organocatalytic literature examples is the use of hydrogen-bonding catalysts, which can activate the imides by hydrogen bonding to both of the carbonyl oxygens.

**Scheme 22:**
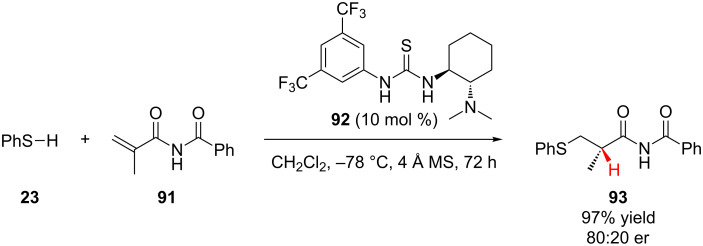
Chen and Wu’s enantioselective addition of thiophenol to *N*-methacryloyl benzamide.

During the course of studying the addition of aromatic thiols to α,β-unsaturated benzamides and enones, Chen, Ding, and Wu first reported the conjugate addition of thiophenol (**23**) to *N*-methacryloyl benzamide **91** followed by enantioselective protonation using Takemoto’s thiourea catalyst **92** in high yield and modest enantioselectivity (Scheme 22) [45].

**Scheme 23:**
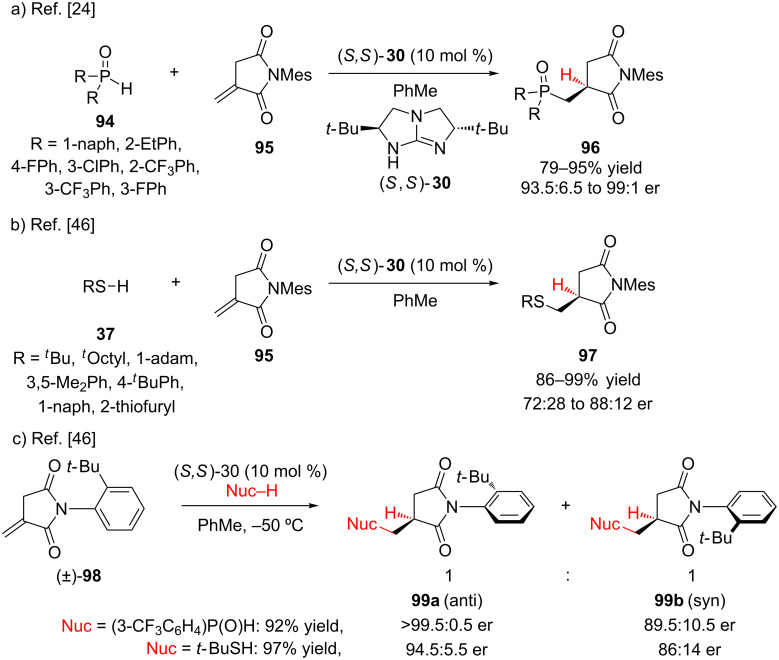
Tan’s enantioselective addition of secondary phosphine oxides and thiols to *N*-arylitaconimides.

Tan and co-workers have investigated the conjugate addition–enantioselective protonation of *N*-arylitaconimides **95** using a *C*_2_-symmetric guanidine catalyst (Scheme 23) [24,46]. Because *E-* and *Z*-enolates can exhibit different enatiofacial selectivity, the use of a cyclic imide ensured exclusive formation of the *Z*-enolate. During optimization, it was found that an *N*-aryl group containing 2,6-disubstitution was crucial for obtaining high levels of enantioselectivity. Addition of a variety of bis-aryl secondary phosphine oxides furnished **96** in high yield and enantioselectivity (Scheme 23a) [24]. Thiols also added efficiently to itaconimide **95** using the same guanidine catalyst system. In general, sterically hindered tertiary thiols added with higher enantioselectivity (85.5:14.5 to 88:22 er) than aromatic thiols (72:28 to 79.5:20.5 er) (Scheme 23b) [46]. This enantioselective addition process could be applied to a racemic mixture of axially chiral *N-*(2-*tert*-butylphenyl)itaconimide (**98**), furnishing atropisomers **99a** and **99b** as a stable and separable 1:1 mixture. A higher enantioselectivity was observed for the *anti*-diastereomer (Scheme 23c).

**Scheme 24:**
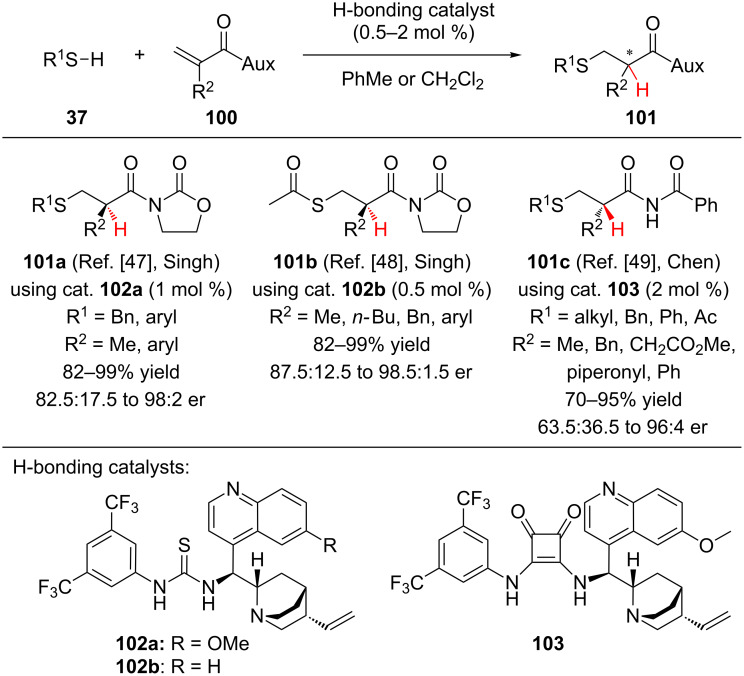
Enantioselective addition of thiols to α-substituted *N*-acryloylamides.

Following Tan’s work on the conjugate addition–enantioselective protonation of cyclic itaconimide **95**, both the Singh and Chen groups investigated the addition of thiol nucleophiles to acyclic imides. Utilizing thiourea catalyst **102a**, Singh and co-workers reported the catalytic enantioselective addition of thiols to *N*-acryloyloxazolidinones, accessing **101a** (Scheme 24) [47]. A variety of electron-rich, neutral, and electron-poor thiols were efficiently coupled under the reaction conditions, including unprotected 2-aminobenzenethiol, although with lower enantioselectivity (82.5:17.5 er). Building on their addition of benzylic and aromatic thiols to *N*-acryloyloxazolidinones, Singh and colleagues demonstrated that thiourea **102b** catalyzes the addition of thioacetic acid to *N*-acryloyloxazolidinones in high yields and enantioselectivity to provide thioester **101b** [48].

In a related transformation, Chen and co-workers showed that squaramide **103** catalyzes the addition of thiols to *N*-acryloylbenzamides to access thioether **101c** [49]. The authors found that exclusion of water by addition of 4 Å molecular sieves as a desiccant was essential for achieving good enantioselectivity. When the R^2^ substituent was aliphatic, good enantioselectivity was observed (87.5:12.5 to 96:4 er); however, aromatic and piperonyl groups greatly diminished the enantioselectivity of the transformation (63.5:36.5 to 73:27 er).

### α,β-Unsaturated ketones

#### Lewis acids

**Scheme 25:**
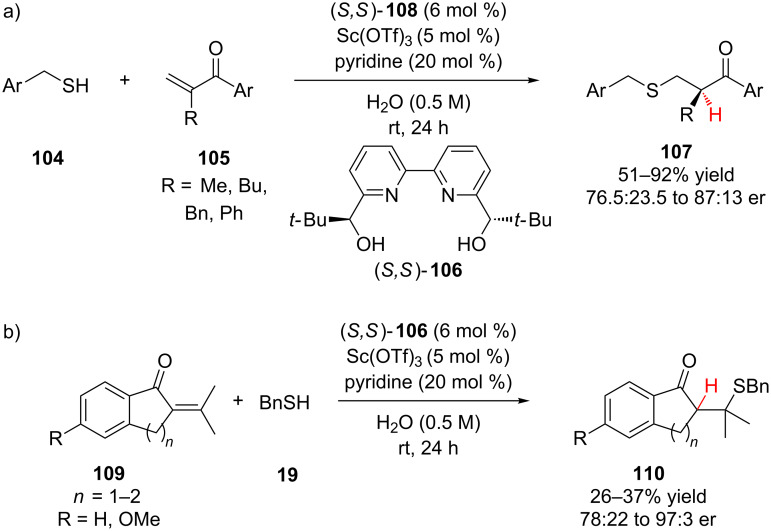
Kobayashi’s enantioselective addition of thiols to α,β-unsaturated ketones.

In 2012, Kobayashi and co-workers reported the Sc-catalyzed enantioselective addition of benzylic thiols **104** to α,β-unsaturated ketones **105** (Scheme 25a) [50]. Interestingly, water was found to be the optimal solvent for this transformation, switching to organic solvents (CH_2_Cl_2_, THF, toluene) resulted in diminished reactivity and poor enantioselectivity. Solvent mixtures of water and THF or ethanol also provided poor reactivity and enantioselectivity. A variety of electron-rich and electron-poor aryl ketones and benzylic thiols were effective substrates. The authors observed that a fast racemic background reaction proceeded in the presence of pyridine. However, in the absence of pyridine the reaction proceeded significantly slower and all enantioselectivity was lost, suggesting the importance of a thiolate intermediate in the catalytic cycle. To suppress the undesired base-catalyzed racemic pathways, β,β-dimethyl substituted tetralone (*n* = 2) and indanone (*n* = 1) substrates **109** were investigated (Scheme 25b). While these substrates displayed diminished reactivity, the tetralones showed a significant boost in enantioselectivity (96:4 to 97:3 er), though only moderate enantioselectivity was observed for the indanone substrate (R = H, 78:22 er).

**Scheme 26:**
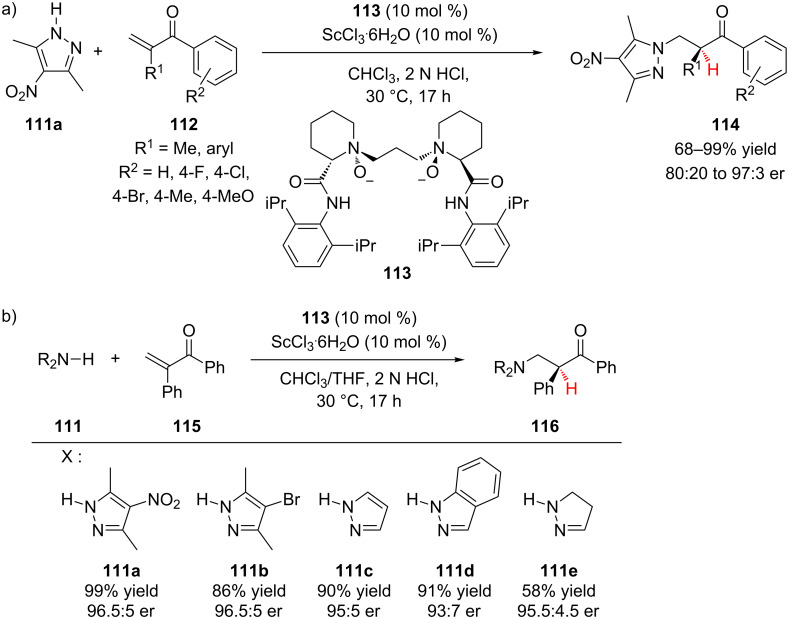
Feng’s enantioselective addition of pyrazoles to α-substituted vinyl ketones.

The Feng group has reported the conjugate addition of pyrazoles to α-substituted aromatic vinyl ketones followed by enantioselective protonation of the enolate intermediate using an *N,N’*-dioxide–Sc(III) catalyst system (Scheme 26) [51]. During optimization, the authors found that incorporation of bulky amides into ligand **113** was crucial for achieving high enantioselectivity. A wide variety of α-aryl vinyl ketones **112** were effective substrates, and only when R^1^ was methyl did the enantioselectivity drop below 92:8 er to 80:20 er (Scheme 26a). The authors also investigated a number of pyrazole nucleophiles **111**, which all reacted with high enantioselectivity, including pyrazoline **111e** (Scheme 26b).

#### Organocatalysts

**Scheme 27:**
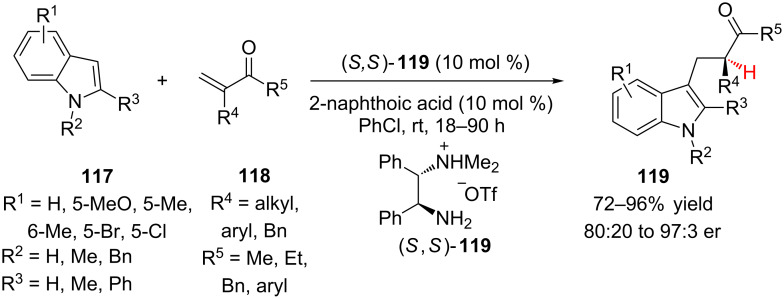
Luo and Cheng’s addition of indoles to vinyl ketones by enamine catalysis.

Building on their earlier work with α,β-unsaturated aldehydes (vide infra), Luo and Cheng have extensively explored the use of enamine catalysis for conjugate addition–enantioselective protonation of vinyl ketones. Using primary amine catalyst (*S,S*)-**119**, the authors were able to catalyze the Friedel–Crafts addition of indoles **117** to vinyl ketones **118** followed by enantioselective protonation (Scheme 27) [52]. During optimization it was found that addition of a weak acid, 2-naphthoic acid, improved both the yield and enantioselectivity of the transformation by facilitating the formation of the iminium ion intermediates. A variety of vinyl ketones **118** were explored for the reaction, and when R^4^ was benzylic, shorter reaction times could be employed (41–48 h) and higher yields and enantioselectivities were observed (78–86% yield, 93:7 to 97:3 er). Aromatic vinyl ketones were also reactive, but required higher temperatures (40–60 ºC). Various indoles **117** were investigated, and although substitution on both the aromatic ring and nitrogen were accommodated, when R^3^ was H, lower enantioselectivity was observed (86.5:13.5 to 89:11 er).

**Scheme 28:**
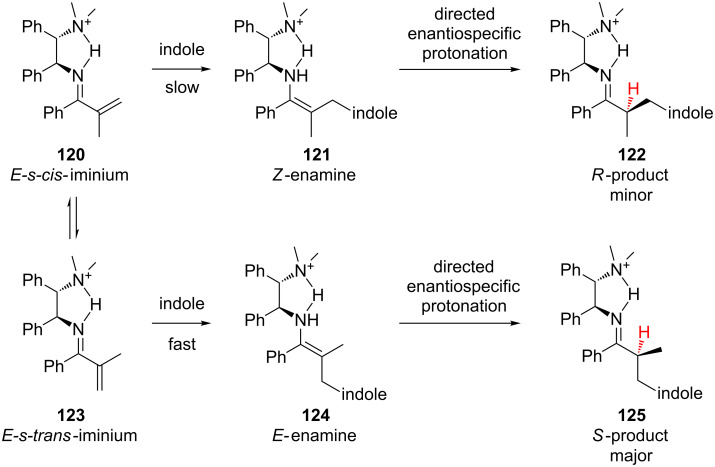
Curtin–Hammett controlled enantioselective addition of indole.

Luo and Cheng also explored the mechanism of the Friedel–Crafts addition of indole to α-substituted vinyl ketones [52–53]. Based on DFT studies, the authors proposed that the stereoselectivity of the reaction was under Curtin–Hammett control (Scheme 28). During the reaction, the iminium ions rapidly interconvert via single-bond rotation. After irreversible C–C bond formation the *E-s-cis*- and *E-s-trans*-iminiums give rise to the *Z-* and *E-*enamines, respectively, which undergo a directed enantiospecific protonation to give either the *R-* or *S-*product. Thus, the enantioselectivity is determined by the ratio of *Z-* to *E*-enamine, which in turn depends on the activation energy difference in the irreversible C–C bond forming steps.

**Scheme 29:**
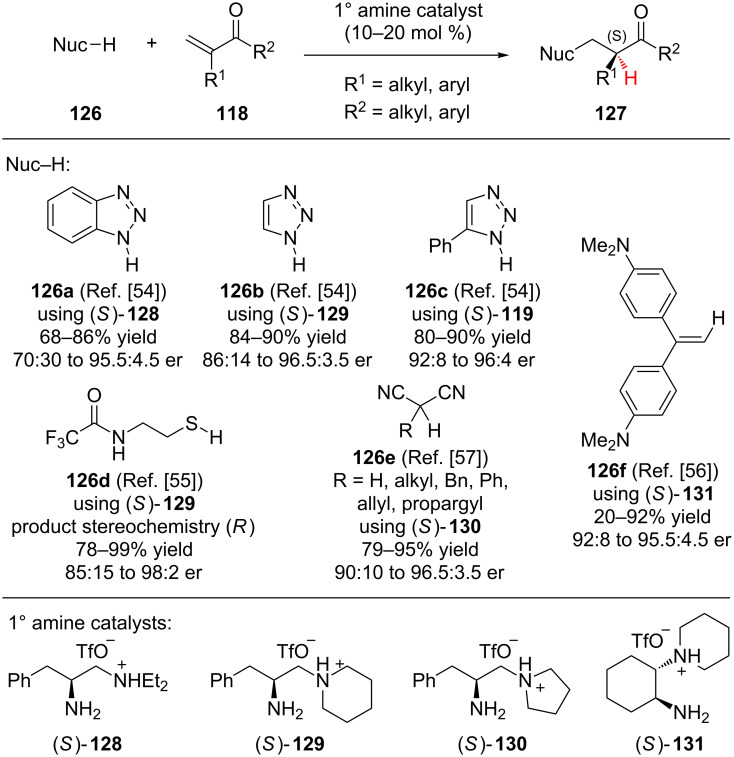
Luo and Cheng’s enantioselective additions to α-branched vinyl ketones.

Having developed an enamine catalysis system where the iminium’s conformation in the conjugate addition step determines the enantioselectivity of the reaction, Lou and Cheng explored the addition of various nitrogen [54], sulfur [55], and carbon nucleophiles [56–57] to α-branched vinyl ketones (Scheme 29). The nitrogen heterocycles benzotriazole (**126a**), triazole (**126b**), and 5-phenyltriazole (**126c**), all reacted smoothly with vinyl ketone **118**. To achieve optimal reactivity and selectivity, each nucleophile required a slightly different chiral amine catalyst. Interestingly, vinylaniline **126f** could also be added to enone **118** in moderate to good yield and with good enantioselectivity. *N-*Trifluoroacetyl-1,2-aminothiol (**126d**) reacted to give thioether products in high yield and enantioselectivity. Only when R^1^ was phenyl did the enantioselectivity of the thiol addition drop below 91.5:8.5 er to 85:15 er. The addition of a variety of α-substituted malononitriles **126e** also proceeded in high yield and enantioselectivity. During the exploration of different nucleophiles some common selectivity trends were observed. Aromatic enones (R^2^ = aryl) generally provided higher levels of enantioselectivity than aliphatic enones (R^2^ = alkyl). Additionally, for all of the nucleophiles, except thiols, increasing the steric bulk at R^1^ resulted in diminished enantioselectivity.

**Scheme 30:**
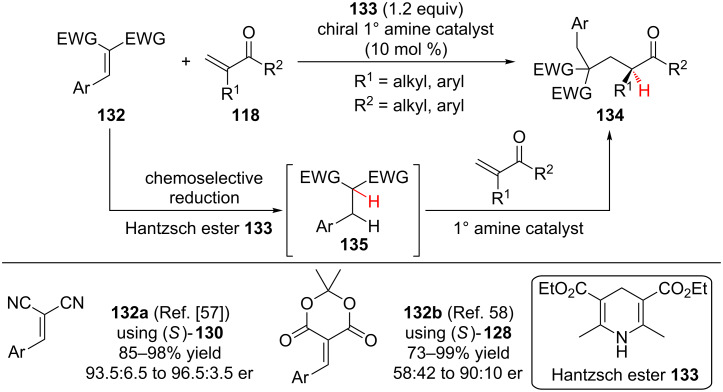
Lou’s reduction–conjugate addition–enantioselective protonation.

Building on his asymmetric conjugate additions to α-substituted enones, Luo demonstrated that Hantzsch ester **133** chemoselectively reduces activated alkenes **132** in the presence of α-substituted vinyl ketones **118**, generating carbon nucleophiles **135** in situ. These nucleophiles then reacted with the α-substituted vinyl ketones **118** via an enamine catalysis conjugate addition–enantioselective protonation pathway (Scheme 30). Lou first applied this strategy of reduction–conjugate addition–enantioselective protonation to α,β-unsaturated malononitriles **132a**, to provide the product in comparable yield and enantioselectivity as when the enantioselective conjugate addition was performed using α-substituted malononitriles (**126e**, Scheme 29) [57]. In a subsequent report, Lou and co-workers demonstrated that Meldrum’s acid derivatives **132b** were also operative in the reduction–conjugate addition–enantioselective protonation pathway [58]. Aromatic α-methyl enones (R^1^ = Me, R^2^ = aryl) reacted in good yield and enantioselectivity. However, when larger R^1^ substituents or an α-methyl enone were used in the reaction, the product was obtained with diminished enantioselectivity (58:42 to 69.5:30.5 er).

After the submission of this review, Lou and co-workers reported an additional example of conjugate addition–enantioselective protonation involving an α,β-unsaturated ketone. In the new report, a chiral primary amine catalyzed conjugate addition of a 1,3-diketone nucleophile into an in situ generated *ortho*-quinone methide was followed by an enamine based retro-Claisen reaction. The resulting enamine was stereoselectively protonated to access α-tertiary alkylated ketones [59].

### α,β-Unsaturated aldehydes

#### Organocatalysts

**Scheme 31:**
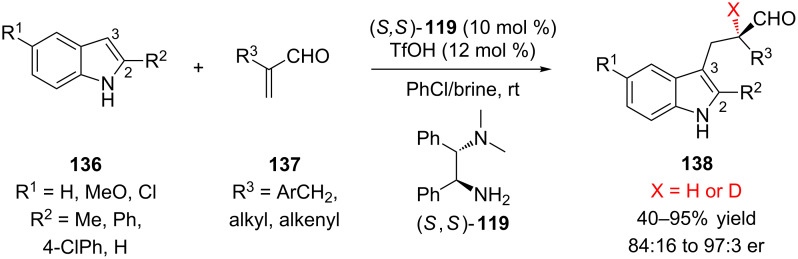
Luo and Cheng’s primary amine-catalyzed addition of indoles to α-substituted acroleins.

In 2011, Luo and Cheng reported the first example of conjugate addition followed by enantioselective protonation using enamine catalysis [60]. Two challenges to using enamine catalysis for enantioselective protonation are the many conformations the reactive iminium intermediates can adopt and the potential for racemization of the enantioenriched product by the basic catalyst. Using the triflate salt of diamine **119**, Luo and Cheng were able to catalyze an enantioselective Friedel–Crafts reaction between indole **136** and α-substituted acrolein **137** (Scheme 31). Acroleins containing a benzyl α-substituent performed best in the reaction with 2-methylindole (72–95% yield, 93.5:6.5 to 97:3 er). Alkyl and alkenyl substituted acroleins also reacted with 2-methylindole, but resulted in lower yields and diminished enantioselectivity (52–80% yield, 87.5:12.5 to 94.5:95.5 er). Electron-rich, neutral, and electron-poor indoles were effective substrates, provided that they contained an R^2^ substituent. The use of indoles unsubstituted at the 2-position (R^2^ = H) led to lower enanantioselectivity (84:16 to 87:13 er). Products were not observed when either α-heteroatom-substituted acroleins or 3-substituted indoles were used as starting materials. When either the enantioenriched or racemic products **138** were resubmitted to the reaction conditions their enantiomeric ratios did not change, indicating that the reaction is not reversible.

**Scheme 32:**
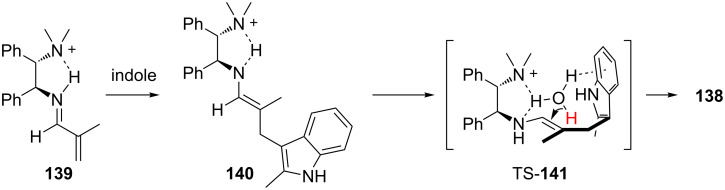
Luo and Cheng’s proposed mechanism and transition state.

During reaction optimization the authors found that the addition of a large excess of brine improved the enantioselectivity of the transformation. Additionally, they found that by using saturated NaCl/D_2_O, α-deuterated products could be obtained in similar enantiomeric ratios as the α-protonated products. Luo and Cheng went on to further investigate this reaction using kinetic studies and DFT calculations [53,60]. Based on their studies, they proposed TS-**141** involving a water molecule stabilized by an O–H/π interaction with the indole ring to explain the improvement in enantioselectivity of the protonation step when brine is used as an additive (Scheme 32).

### α,β-Unsaturated thioesters

#### Lewis acids

**Figure 3:**
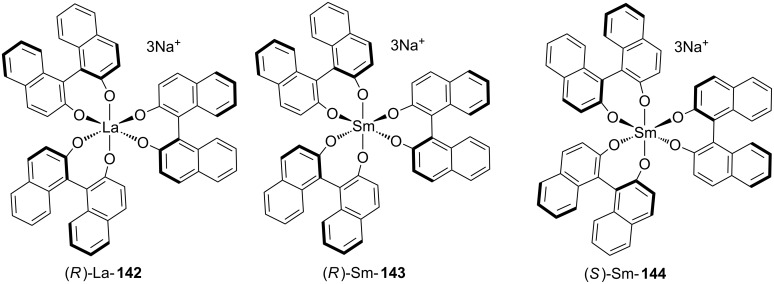
Shibasaki’s chiral lanthanum and samarium tris(BINOL) catalysts.

**Scheme 33:**
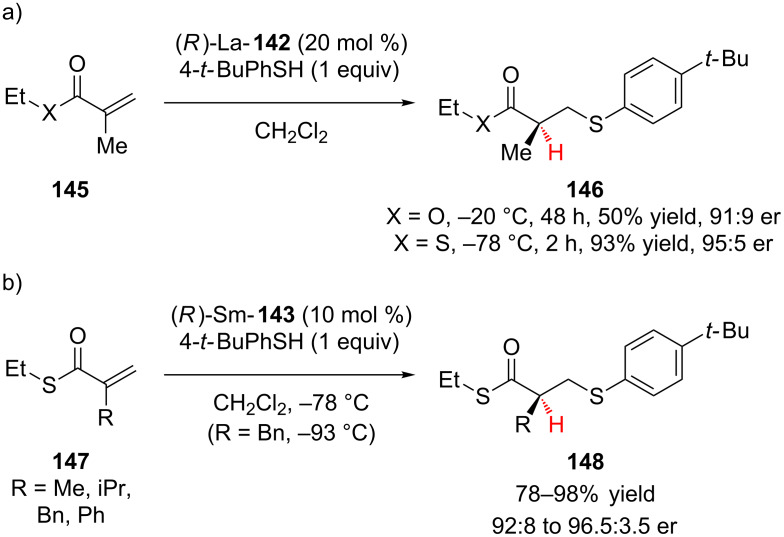
Shibasaki’s enantioselective addition of 4-*tert*-butyl(thiophenol) to α,β-unsaturated thioesters.

In one of the early examples of conjugate addition–enantioselective protonation, Shibasaki and co-workers demonstrated that chiral lanthanum and samarium tris(BINOL) complexes (Figure 3), developed by the Shibasaki group for asymmetric Michael additions using malonates and organometallic reagents, are effective catalysts for the sequential conjugate addition of 4-*tert*-butyl(thiophenol) to α,β-unsaturated thioesters followed by enantioselective protonation (Scheme 33) [61].

During their initial investigations Shibasaki and co-workers examined the (*R*)-La-**142** catalyzed enantioselective addition of 4-*tert*-butyl(thiophenol) to ethyl methacrylate **145** (X = O) (Scheme 33a), while good enantioselectivity could be achieved, the saturated ester product **146** could not be obtained in greater than 50% yield. In contrast, the unsaturated thioester **145** (X = S) provided the product in high yield and enantioselectivity. By switching to samarium catalyst (*R*)-Sm-**143**, the catalyst loading could be halved and a further improvement in enantioselectivity was observed (Scheme 33b). For example, for R = Me an 86% yield and 96.5:3.5 er was obtained [61].

**Scheme 34:**
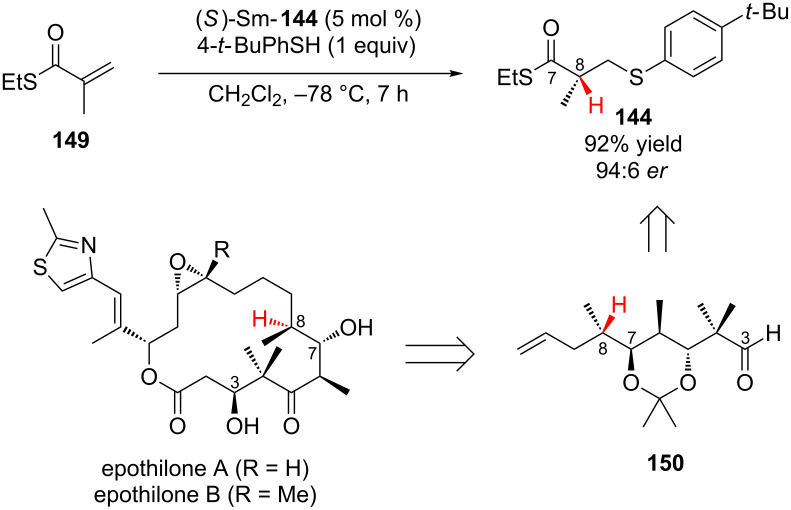
Shibasaki’s application of chiral (*S*)-SmNa_3_tris(binaphthoxide) catalyst **144** to the total synthesis of epothilones A and B.

The Shibasaki group went on to apply their conjugate addition–enantioselective protonation of α,β-unsaturated thioesters to the total synthesis of epothilones A and B, natural products that inhibit microtubule function [62]. The enantioselective addition of 4-*tert*-butyl(thiophenol) to **149** using 5 mol % of (*S*)-Sm-**144** was used to set the C8 stereocenter in the final product (Scheme 34).

### α,β-Unsaturated amides

#### Lewis acids

**Scheme 35:**
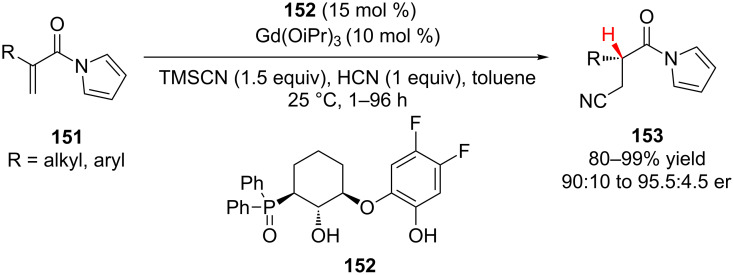
Shibasaki’s cyanation–enantioselective protonation of *N*-acylpyrroles.

In 2009, the Shibasaki group reported the catalytic cyanation–enantioselective protonation of α-substituted α,β-unsaturated *N*-acylpyrroles **151** using a chiral polynuclear Gd complex (Scheme 35) [63]. Using a gadolinium catalyst derived from tridentate ligand **152**, conjugate addition of cyanide followed by enantioselective protonation occurred with high yield and enantioselectivity for both α-alkyl and α-aryl conjugate addition acceptors. Alkyl substrates reacted smoothly at 25 °C (90–98% yield, 90:10 to 95.5-4.5 er) even when R was a more sterically demanding isopropyl or cyclohexyl group. Aryl substrates required lower reaction temperatures and longer reaction times (−30 or −78 °C). The average yield and enantioselectivity for the aryl substrates was lower than for the alkyl substrates; however, many of the aryl substituted products were crystalline and could be recrystallized up to greater than 98.5:1.5 er. Interestingly, when attempting to use catalytic amounts of trimethylsilyl cyanide (10 mol %) a significant decrease in reaction rate and enantioselectivity was observed.

#### Transition metal catalysts

**Scheme 36:**
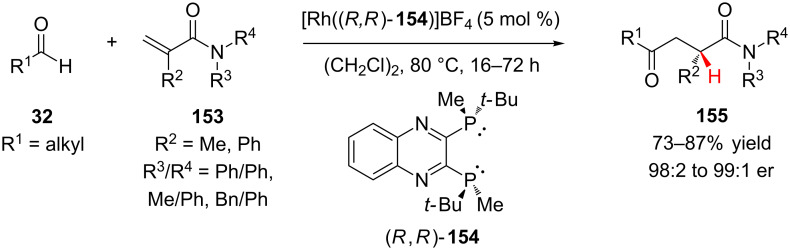
Tanaka’s hydroacylation of acrylamides with aliphatic aldehydes.

Conjugate addition–enantioselective protonation of α,β-unsaturated tertiary amides can also be performed with transition metal catalysts. Using a cationic rhodium(I)/QuinoxP* **154** complex, Tanaka and co-workers achieved the enantioselective hydroacylation of α-substituted acrylamides **153** using aliphatic aldehydes **32** to provide 1,4-ketoamides **155** in high yields and excellent enantioselectivity (Scheme 36) [64]. A variety of aliphatic aldehydes were effective substrates, including the α-branched cyclohexyl and cyclopentyl carboxaldehydes. However, pivaldehyde was completely unreactive, and when benzaldehyde was examined as a substrate, the reaction was slow and the enantioselectivity was poor (12% yield and 79:21 er).

### α,β-Unsaturated nitroalkenes

#### Organocatalysts

**Scheme 37:**
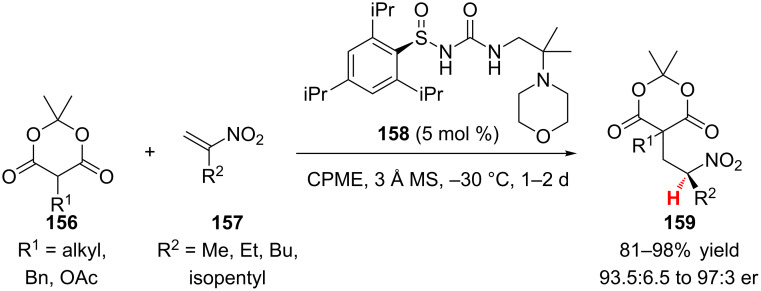
Ellman’s enantioselective addition of α-substituted Meldrum’s acids to terminally unsubstituted nitroalkenes.

In the first reported example of the enantioselective protonation of a nitronate, Ellman and co-workers demonstrated that an *N*-sulfinylurea organocatalyst could be used to catalyze the addition of α-substituted Meldrum’s acids to terminally unsubstituted nitroalkenes (Scheme 37) [65]. Interestingly, the optimal organocatalyst for the transformation was chiral only at sulfur, when chiral amine motifs were explored, e.g., 1,2-cyclohexanediamine, poor enantioselectivity was observed (≤75:25 er). A variety of R^1^ substituents on Meldrum’s acid **156** were compatible with the reaction, including alkyl, phenethyl, pendant esters and thioethers, and an α-acetyloxy group (81–98% yield, 95.5:4.5 to 97:3 er). Slightly lower enantioselectivity was observed only when R^1^ was benzyl (93.5:6.5 er). Methyl, ethyl, *n*-butyl, and isopentyl R^2^ substituted nitroalkenes were efficient substrates (84–98% yield, 95.5:4.5 to 97:3 er). The resulting nitroalkane adduct **159** could be reduced and cyclized to access α,γ-disubstituted γ-lactams.

**Scheme 38:**
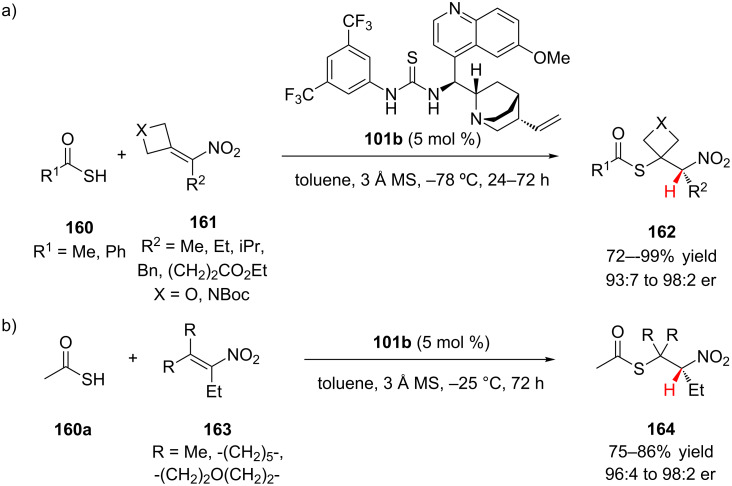
Ellman’s enantioselective addition of thioacids to α,β,β-trisubstituted nitroalkenes.

Ellman and co-workers, in the second example of conjugate addition–enantioselective protonation with nitroalkenes, showed that thioacids **160** could be added in high yields and with high enantioselectivity to α,β,β-trisubstituted nitroalkenes **161** using a thiourea organocatalyst **101b** (Scheme 38) [66]. This report was the first example of enantioselective addition to a trisubstituted nitroalkene and was the first example of conjugate addition–enantioselective protonation using a fully substituted alkene. Nitroalkenes that were activated by the incorporation of an oxetane or *N-*Boc-azetidine ring at the β-position reacted well with both thioacetic acid and thiobenzoic acid (Scheme 38a). Various R^2^ substituents were compatible with the reaction, including an isopropyl group and a pendent methyl ester. Generally, the azetidine nitroalkenes provided the 1,2-nitrothioacetates in higher yields and enantioselectivity (81–99% yield, 95:5 to 98:2 er). The oxetane and *N-*Boc azetidine nitroalkenes were activated toward conjugate addition by the release of ring-strain. However, thioacetic acid (**160a**) also adds in good yield and high enantioselectivity to unstrained nitroalkenes **163** (Scheme 38b). Additions to β-cyclohexyl and β-4-tetrahydropyran nitroalkenes as well as an acyclic β,β-dimethyl nitroalkene all proceeded with good conversion when the reaction temperature was raised to −25 °C.

After the submission of this review, Ellman and co-workers published an additional example of enantioselective nitronate protonation, which was accomplished using an *N*-sulfinylurea organocatalyst to catalyze the addition of a pyrazol-5-one nucleophile to a trisubstituted nitroalkene [67].

### α,β-Unsaturated phosphonates and phosphine oxides

#### Transition metal catalysts

**Scheme 39:**
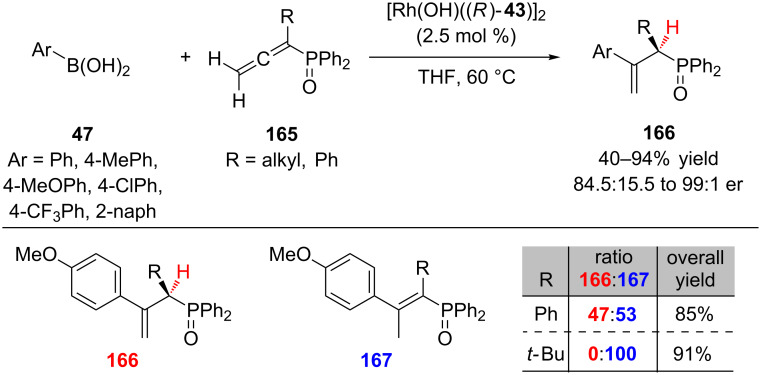
Hayashi’s enantioselective hydroarylation of diphenylphosphinylallenes.

In 2006, Hayashi and co-workers reported the first conjugate addition–enantioselective protonation of allenes **165** bearing a phosphine oxide (Scheme 39) [68]. Incorporation of the phosphine oxide allowed for selective protonation forming the less stabilized terminal chiral alkene **166** over the internal achiral isomer **167**, an inherent challenge in the hydroarylation of terminal allenes. Electron-rich, neutral, and electron-poor arylboronic acids **47** added to alkyl (R = Me, Et, *n*-Bu) diphenylphosphinylallenes **165** in high yield and excellent enantioselectivity (85–94% yield, 98:2 to 99:1 er). With sterically bulky α-substituents (R = Ph, *t*-Bu), competitive formation of the achiral internal alkene was preferred.

**Scheme 40:**
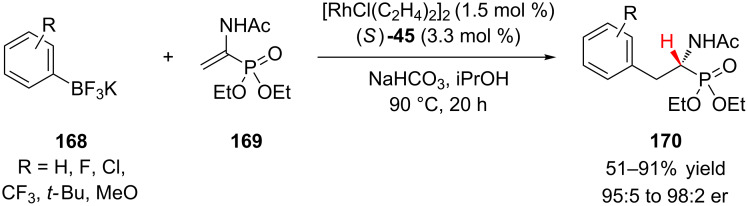
Hayashi’s enantioselective hydroarylation of diphenylphosphinylallenes.

Enantioenriched α-amino phosphonic acids and their derivatives are important motifs that have been utilized by the pharmaceutical and agrochemical industries as α-amino acid analogues. Darses and co-workers reported a novel approach to the synthesis of enantioenriched α-amino phosphonates **170** via a rhodium(I) catalyzed enantioselective 1,4-addition of potassium aryltrifluoroborates **168** to dehydroaminophosphonates **169** (Scheme 40) [69]. α-Amino phosphonates **170** were obtained with high levels of enantioselectivity for electron-rich, neutral, and electron-poor aryltrifluoroborates **168**. Electron-rich organoboron reagents (4-*t*-Bu, 4-MeO, and 3-MeO) gave slightly lower yields (51–77%) than the electron-poor reagents (65–86%), with potassium phenyltrifluoroborate giving the highest yield (91%). Phenylboronic acid was also shown to be a competent coupling partner, providing the corresponding product in 92% yield and 96.5:3.5 er.

### Methacrylonitrile

#### Lewis acids

**Figure 4:**
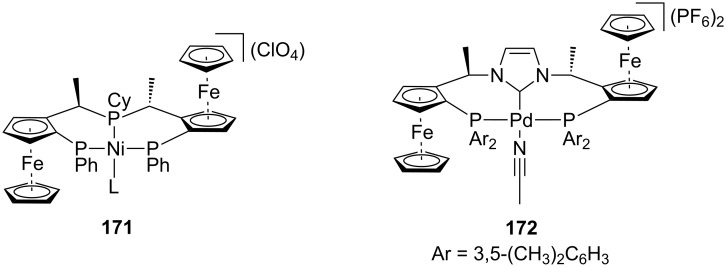
Togni’s chiral ferrocenyl tridentate nickel(II) and palladium(II) complexes.

In the literature, conjugate addition–enantioselective protonation using α,β-unsaturated nitriles has remained unexplored for substrates other than methacrylonitrile. The Togni lab has explored using ferrocenyl tridentate nickel(II) and palladium(II) complexes as chiral Lewis acid catalysts for the hydrophosphination and hydroamination of methacrylonitrile (Figure 4) [70–74]. Other researchers have reported platinum [75], nickel [76], and zirconium [77] catalysts for the hydrophosphination and hydroamination of methacrylonitrile; however, these examples provided product with significantly lower enantioselectivity.

**Scheme 41:**
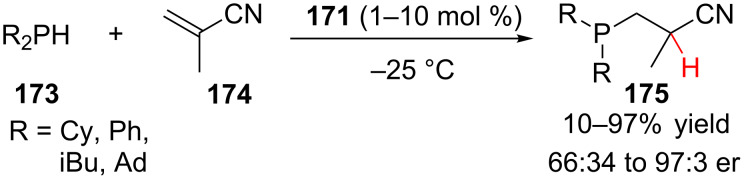
Togni’s enantioselective hydrophosphination of methacrylonitrile.

In 2004, the Togni lab reported the hydrophosphination of methacrylonitirle **174** catalyzed by nickel(II)/(*R,R*)-Pigiphos complex **171** (Scheme 41) [71]. The reaction proceeded in good yield for alkyl phosphines (71–97% yield) and with higher enantioselectivity for the more sterically encumbered alkyl groups (R = Ad, 97:3 er).

**Scheme 42:**
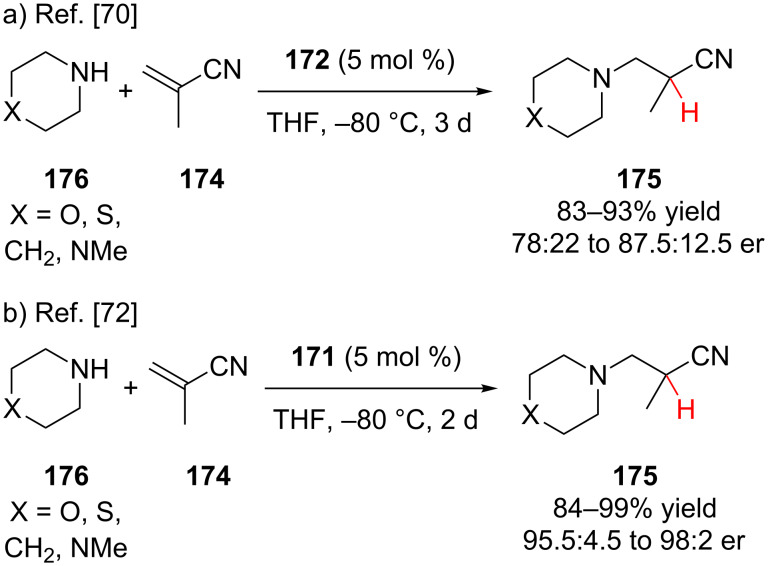
Togni’s enantioselective hydroamination of methacrylonitrile.

Togni and co-workers subsequently reported the hydroamination of methacrylonitrile **174** using palladium(II) catalyst **172** (Scheme 42a) [72]. Low temperature and bulky aryl groups on the catalyst were necessary to achieve good levels of enantioselectivity. In a later study, their nickel(II) catalyst **171** proved to be much more selective under the same reaction conditions (Scheme 42b) [74]. Togni and co-workers also investigated the hydroamination of methacrylonitrile using benzylamine and aniline; however, these substrates gave products with low enantioselectivity (55:45 to 61:29 er).

## Conclusion

As described above, many conjugate addition–enantioselective protonation reactions have been employed for the synthesis of enantioenriched amino esters and other carboxylic acid derivatives. Additions to α,β-unsaturated esters and imides have historically been the most extensively investigated substrates with additions to other classes of Michael acceptors only being reported more recently. While many examples using sulfur and carbon nucleophiles have been reported, the addition of other heteroatom nucleophiles remains relatively unexplored. This approach for the synthesis of tertiary carbon stereocenters continues to generate interest, both for the challenges the transformation presents and the ability to efficiently access useful motifs.

## References

[1] Fehr C (1996). Angew Chem, Int Ed Engl.

[2] Yanagisawa A, Ishihara K, Yamamoto H (1997). Synlett.

[3] Eames J, Weerasooriya N (2001). Tetrahedron: Asymmetry.

[4] Duhamel L, Duhamel P, Plaquevent J-C (2004). Tetrahedron: Asymmetry.

[5] Mohr J T, Hong A Y, Stoltz B M (2009). Nat Chem.

[6] Poisson T, Kobayashi S, Andrushko V, Andrushko N (2013). Asymmetric Protonation of Carbanions and Polar Double Bonds: Application to Total Syntheses. Stereoselective Synthesis of Drugs and Natural Products.

[7] Wang C, Goh C M T, Xiao S, Ye W, Tan C-H (2013). J Synth Org Chem, Jpn.

[8] Leow D, Shen J, Su Y, Peh G (2014). Mini-Rev Org Chem.

[9] Oudeyer S, Brière J-F, Levacher V (2014). Eur J Org Chem.

[10] Frongia A, Secci F, Melis N (2015). C R Chim.

[11] Church T L, Andersson P G (2008). Coord Chem Rev.

[12] Khumsubdee S, Burgess K (2013). ACS Catal.

[13] Verendel J J, Pámies O, Diéguez M, Andersson P G (2014). Chem Rev.

[14] Nishimura K, Ono M, Nagaoka Y, Tomioka K (2001). Angew Chem, Int Ed.

[15] Sibi M P, Asano Y, Sausker J B (2001). Angew Chem, Int Ed.

[16] Sibi M P, Patil K (2004). Angew Chem, Int Ed.

[17] Sibi M P, Patil K (2005). Org Lett.

[18] Yajima T, Tonoi T, Nagano H, Tomita Y, Mikami K (2010). Eur J Org Chem.

[19] Kieffer M E, Repka L M, Reisman S E (2012). J Am Chem Soc.

[20] Ishihara K, Nakamura S, Kaneeda M, Yamamoto H (1996). J Am Chem Soc.

[21] Nakamura S, Kaneeda M, Ishihara K, Yamamoto H (2000). J Am Chem Soc.

[22] Pracejus H, Wilcke F-W, Hanemann K (1977). J Prakt Chem.

[23] Kumar A, Salunkhe R V, Rane R A, Dike S Y (1991). J Chem Soc, Chem Commun.

[24] Leow D, Lin S, Chittimalla S K, Fu X, Tan C-H (2008). Angew Chem, Int Ed.

[25] Jousseaume T, Wurz N E, Glorius F (2011). Angew Chem, Int Ed.

[26] Wurz N E, Daniliuc C G, Glorius F (2012). Chem – Eur J.

[27] Kuniyil R, Sunoj R B (2013). Org Lett.

[28] Farley A J M, Sandford C, Dixon D J (2015). J Am Chem Soc.

[29] Reetz M T, Moulin D, Gosberg A (2001). Org Lett.

[30] Chapman C J, Wadsworth K J, Frost C G (2003). J Organomet Chem.

[31] Moss R J, Wadsworth K J, Chapman C J, Frost C G (2004). Chem Commun.

[32] Hargrave J D, Herbert J, Bish G, Frost C G (2006). Org Biomol Chem.

[33] Navarre L, Darses S, Genet J-P (2004). Angew Chem, Int Ed.

[34] Navarre L, Martinez R, Genet J-P, Darses S (2008). J Am Chem Soc.

[35] Sibi M P, Tatamidani H, Patil K (2005). Org Lett.

[36] Frost C G, Penrose S D, Lambshead K, Raithby P R, Warren J E, Gleave R (2007). Org Lett.

[37] Filloux C M, Rovis T (2015). J Am Chem Soc.

[38] Hamashima Y, Somei H, Shimura Y, Tamura T, Sodeoka M (2004). Org Lett.

[39] Sodeoka M, Hamashima Y, Tamura T, Suzuki S (2009). Synlett.

[40] Hamashima Y, Suzuki S, Tamura T, Somei H, Sodeoka M (2011). Chem – Asian J.

[41] Phua P H, Mathew S P, White A J P, de Vries J G, Blackmond D G, Hii K K (2007). Chem – Eur J.

[42] Reboule I, Gil R, Collin J (2008). Eur J Org Chem.

[43] Sibi M P, Coulomb J, Stanley L M (2008). Angew Chem, Int Ed.

[44] Poisson T, Yamashita Y, Kobayashi S (2010). J Am Chem Soc.

[45] Li B-J, Jiang L, Liu M, Chen Y-C, Ding L-S, Wu Y (2005). Synlett.

[46] Lin S, Leow D, Huang K-W, Tan C-H (2009). Chem – Asian J.

[47] Rana N K, Singh V K (2011). Org Lett.

[48] Unhale R A, Rana N K, Singh V K (2013). Tetrahedron Lett.

[49] Chen F, Dai L, Yang H, Niu J (2012). Synlett.

[50] Kitanosono T, Sakai M, Ueno M, Kobayashi S (2012). Org Biomol Chem.

[51] Zhang J, Zhang Y, Liu X, Guo J, Cao W, Lin L, Feng X (2014). Adv Synth Catal.

[52] Fu N, Zhang L, Luo S, Cheng J-P (2013). Chem – Eur J.

[53] Zhang L, Fu N, Luo S (2015). Acc Chem Res.

[54] Fu N, Zhang L, Luo S, Cheng J-P (2014). Org Chem Front.

[55] Fu N, Zhang L, Luo S, Cheng J-P (2014). Org Lett.

[56] Cui L, Zhang L, Luo S, Cheng J-P (2014). Eur J Org Chem.

[57] Fu N, Zhang L, Luo S (2015). Org Lett.

[58] Luo S, Fu N, Guo Y, Zhang L (2015). Synthesis.

[59] Zhu Y, Zhang L, Luo S (2016). J Am Chem Soc.

[60] Fu N, Zhang L, Li J, Luo S, Cheng J-P (2011). Angew Chem, Int Ed.

[61] Emori E, Arai T, Sasai H, Shibasaki M (1998). J Am Chem Soc.

[62] Sawada D, Kanai M, Shibasaki M (2000). J Am Chem Soc.

[63] Morita M, Drouin L, Motoki R, Kimura Y, Fujimori I, Kanai M, Shibasaki M (2009). J Am Chem Soc.

[64] Shibata Y, Tanaka K (2009). J Am Chem Soc.

[65] Kimmel K L, Weaver J D, Lee M, Ellman J A (2012). J Am Chem Soc.

[66] Phelan J P, Patel E J, Ellman J A (2014). Angew Chem, Int Ed.

[67] Phelan J P, Ellman J A (2016). Adv Synth Catal.

[68] Nishimura T, Hirabayashi S, Yasuhara Y, Hayashi T (2006). J Am Chem Soc.

[69] Lefevre N, Brayer J-L, Folléas B, Darses S (2013). Org Lett.

[70] Gischig S, Togni A (2004). Organometallics.

[71] Sadow A D, Haller I, Fadini L, Togni A (2004). J Am Chem Soc.

[72] Gischig S, Togni A (2005). Eur J Inorg Chem.

[73] Fadini L, Togni A (2007). Helv Chim Acta.

[74] Fadini L, Togni A (2008). Tetrahedron: Asymmetry.

[75] Kovacik I, Wicht D K, Grewal N S, Glueck D S, Incarvito C D, Guzei I A, Rheingold A L (2000). Organometallics.

[76] Yang Z, Liu D, Liu Y, Sugiya M, Imamoto T, Zhang W (2015). Organometallics.

[77] El-Zoghbi I, Kebdani M, Whitehorne T J J, Schaper F (2013). Organometallics.

